# On-Demand Patient-Specific Phenotype-to-Genotype Ebola Virus Characterization

**DOI:** 10.3390/v13102010

**Published:** 2021-10-06

**Authors:** Brett F. Beitzel, Sheli R. Radoshitzky, Nicholas Di Paola, Jennifer M. Brannan, David Kimmel, Katie Caviness, Veronica Soloveva, Shuiqing Yu, Elena N. Postnikova, Courtney L. Finch, Hu Liu, Laura Prugar, Russell Bakken, John M. Dye, Jeffrey R. Kugelman, James M. Cunningham, Mariano Sanchez-Lockhart, Jens H. Kuhn, Gustavo Palacios

**Affiliations:** 1 United States Army Medical Research Institute of Infectious Diseases (USAMRIID), Fort Detrick, Frederick, MD 21702, USA; bfbeitzel@gmail.com (B.F.B.); sheli.r.radoshitzky.ctr@mail.mil (S.R.R.); nicholas.dipaola.civ@mail.mil (N.D.P.); jennifer.m.brannan.ctr@mail.mil (J.M.B.); david.j.kimmel5.ctr@mail.mil (D.K.); katie.caviness@gmail.com (K.C.); veronica.soloveva.ctr@mail.mil (V.S.); lprugar@gmail.com (L.P.); russell.r.bakken.civ@mail.mil (R.B.); john.m.dye1.civ@mail.mil (J.M.D.); jeffrey.r.kugelman.mil@mail.mil (J.R.K.); marsanlock@gmail.com (M.S.-L.); 2 The Geneva Foundation, Tacoma, WA 98402, USA; 3 Integrated Research Facility at Fort Detrick (IRF-Frederick), National Institute of Allergy and Infectious Diseases (NIAID), National Institutes of Health (NIH), Fort Detrick, Frederick, MD 21702, USA; shuiqing.yu@nih.gov (S.Y.); elena.postnikova2@nih.gov (E.N.P.); courtney.finch@nih.gov (C.L.F.); kuhnjens@mail.nih.gov (J.H.K.); 4 Department of Medicine, Brigham and Women’s Hospital and Department of Microbiology, Harvard Medical School, Boston, MA 02115, USA; chestnuthill2015@gmail.com (H.L.); jcunningham@rics.bwh.harvard.edu (J.M.C.)

**Keywords:** Ebola virus, EBOV, reverse genetics, genotype-to-phenotype, variants, on-demand, patient-specific, synthetic genomics, medical countermeasure

## Abstract

Biosafety, biosecurity, logistical, political, and technical considerations can delay or prevent the wide dissemination of source material containing viable virus from the geographic origin of an outbreak to laboratories involved in developing medical countermeasures (MCMs). However, once virus genome sequence information is available from clinical samples, reverse-genetics systems can be used to generate virus stocks de novo to initiate MCM development. In this study, we developed a reverse-genetics system for natural isolates of Ebola virus (EBOV) variants Makona, Tumba, and Ituri, which have been challenging to obtain. These systems were generated starting solely with in silico genome sequence information and have been used successfully to produce recombinant stocks of each of the viruses for use in MCM testing. The antiviral activity of MCMs targeting viral entry varied depending on the recombinant virus isolate used. Collectively, selecting and synthetically engineering emerging EBOV variants and demonstrating their efficacy against available MCMs will be crucial for answering pressing public health and biosecurity concerns during Ebola disease (EBOD) outbreaks.

## 1. Introduction

Novel Ebola disease (EBOD) cases have sporadically occurred since 2017. A 2018–2020 outbreak in Middle Africa predominantly occurred in remote, conflict-driven areas that complicated and delayed clinical sample acquisition. Reverse-genetic systems can help overcome some of the geographical, political, and logistical constrains associated with obtaining bona fide patient samples, and will be crucial to current and future public health responses. We have recovered isolates of three distinct Ebola virus (EBOV) variants from recent outbreaks, one of which has not been described previously. By generating these isolates de novo, cell culture passage-derived genome mutations that can alter sensitivity and affect therapeutic efficacy of medical countermeasures (MCMs) in vitro are bypassed. We have also demonstrated the capability to evaluate in vivo virulence of different isolates by testing mutants of concern such as A82V. Readily acquiring patient-specific viral isolates of high consequence pathogens, such as EBOV, can be a critical tool to assess medical countermeasure efficacy.

The first documented outbreaks of EBOD occurred in 1976 in southern Sudan (now South Sudan) and northern Zaire (now the Democratic Republic of the Congo [COD]) and were caused by two distinct ebolaviruses, Sudan virus (SUDV) and EBOV, respectively [1,2,3,4,5]. From 1976 to 2013, EBOD cases were detected primarily throughout Middle Africa and Eastern Africa, and the outbreaks were relatively limited in scope—from single cases to 425 cases during a 2000–2001 Sudan virus disease (SVD) outbreak in Uganda [6]. That changed by the end of 2013, however, when EBOV infections occurred in Western Africa: Initial infections in Guinea spread to the neighboring countries of Sierra Leone and Liberia (with small numbers of cases arising in several other countries); ending in 2016, this outbreak caused 28,652 confirmed, probable, and suspected cases and 11,325 deaths [7]. After 2013, five additional EBOV disease (EVD) outbreaks occurred in COD: In 2014, Équateur Province experienced a small outbreak involving 69 cases (and 49 deaths) and another in 2018 with 54 cases (and 33 deaths) [8,9]. In 2017, Bas-Uele Province recorded eight EVD cases (and four deaths) [10]. From mid-2018 to mid-2020, Nord-Kivu Province and Ituri Province experienced the second-largest EVD outbreak on record [11], which resulted in 3481 cases and 2299 deaths [12]. Most recently, from June to November 2020, an EVD outbreak in Équateur Province included 118 confirmed cases and 55 deaths [13].

During and after these outbreaks in Western Africa and COD, a large number of research groups determined and deposited in GenBank close to 2000 complete and nearly complete EBOV genome sequences [8,11,14,15,16,17,18,19,20,21,22,23,24,25]. These sequences revealed several mutations that may have been functionally selected during human-to-human transmission amid outbreaks. The consequences of these mutations on pathogenesis or transmission are largely unknown [26,27,28]. Other identified mutations (often patient-specific) may affect sequence-based diagnostics negatively or alter susceptibility to MCMs [11]. However, due to biosafety, biosecurity, logistical, political, and technical considerations, only a very limited number of natural replicating EBOV isolates were obtained during each outbreak and made available to research groups across borders, thus significantly impeding the study of these isolates. Unsurprisingly, the few isolates that were made available did not necessarily have genomes with mutations of concern. Complicating the issue further, EBOV isolation procedures typically involve serial passaging of an original clinical isolate in cell culture to obtain virus stocks. Such passaging rapidly generates cell-culture-specific genome adaptations that may obfuscate natural mutations and phylogenetic analyses, in particular if the cells used for culturing are derived from animals other than the isolation host (humans) [29,30,31]. Some of these EBOV adaptations may even affect clinical outcomes in experimentally infected animals [32,33].

Reverse-genetics systems for filoviruses were first developed in the early 2000s [34,35]. Using these systems enables recovery of infectious filoviruses starting from a cloned copy of the filoviral genome. To recover infectious viruses, filoviral “helper proteins” must be provided in trans to initiate “illegitimate” encapsidation of the genome, transcription of structural protein messenger RNA (mRNA) from the genomic RNA, and replication of the genome and resulting antigenomes. In the case of EBOV, these helper proteins are the filoviral nucleoprotein (NP), polymerase cofactor (viral protein 35 [VP35]), transcriptional enhancer (VP30), and RNA-directed RNA polymerase activity-encoding large protein (L) [36].

Although numerous filovirus reverse-genetics systems have been developed [37], a majority were established using a consensus filovirus genome sequence that was determined using a cell-cultured filovirus isolate and subsequent cloning of the filovirus genome using reverse transcription polymerase chain reaction (RT-PCR). Despite large case counts, natural EBOV isolates from the 2013–2016 EVD outbreak in Western Africa (EBOV variant Makona [EBOV/Mak]), the 2018 EVD outbreak in COD’s Équateur Province (EBOV variant Tumba [EBOV/Tum]), and the 2018–2020 outbreak in COD’s Nord-Kivu Province and Ituri Province (EBOV variant Ituri [EBOV/Itu]) are difficult to obtain primarily due to biosafety concerns. There are also challenges associated with obtaining even these few isolates via international transfer, prompted us to create isolates of these variants de novo. Our “reverse-genomics” approach uses classic filovirus reverse-genetics techniques but is based on de novo in vitro synthesis of filovirus genomes that does not require genomic RNA extracted from natural virus isolates. A similar approach was recently used by others [38]. This approach is not derived from consensus sequencing but instead is virus genotype-specific, representing a particular filovirus variant “isolate” of a virus population circulating in a particular patient at a particular sampling time point.

In this study, we developed a reverse-genetics system for EBOV/Mak, including three derivatives with mutations of interest, and used these viruses to demonstrate that cell culture adaptations can have profound effects on responses to MCMs. Additionally, we showed that the recombinant rescued viruses behave in a manner similar to that of natural EBOV isolates in a laboratory mouse model of EVD. Finally, we developed a reverse-genetics system for EBOV/Itu and, to our knowledge, the first reported reverse-genetics system for EBOV/Tum. We have successfully generated recombinant viruses from all of these systems, facilitating research on these difficult-to-obtain EBOV variants.

## 2. Materials and Methods

### 2.1. Cell Culture

Cell lines used for virus rescues included human cervix epithelial HeLa cells (American Type Culture Collection [ATCC], Manassas, VA, USA; ATCC CRM-CCL-2), HEK 293T cells (ATCC CRL-11268); human hepatocellular carcinoma epithelial Huh7 cells (Health Science Research Resources Bank [JCRB0403], Osaka, Japan), Huh7D-12/T7 (a Huh7 cell line stably expressing hepatitis delta antigen [HDAg] from Millipore Sigma, St. Louis, MO, USA, modified by Genecopoeia Rockville, MD, USA to stably express T7 RNA polymerase for this study), and grivet kidney epithelial Vero C1008/E6 cells (BEI Resources, Manassas, VA, USA; Catalog Number [Cat. No.] NR596). Cells were maintained in Dulbecco’s Modified Eagle’s Medium (DMEM; Thermo Fisher Scientific, Waltham, MA, USA) +10% heat-inactivated fetal bovine serum (FBS; GE Healthcare Life Sciences, Pittsburgh, PA, USA and Sigma) at 37 °C and 5% carbon dioxide (CO_2_).

### 2.2. Viruses

**Figure 1:**
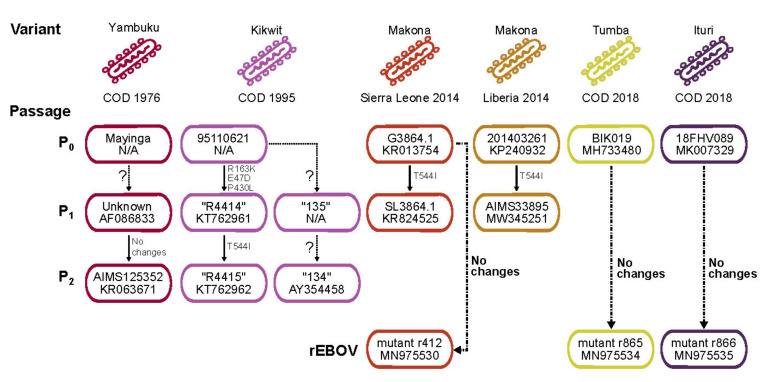
Historical records of natural and synthetic Ebola virus (EBOV) isolates used in this study. EBOV variants are uniquely colored; country of origin and year of collection are shown. Passage information is provided: P0 indicates the natural isolate; P1 and P2 indicate subsequent passages in model organisms. An identifier and GenBank accession number, if available, is provided. The question marks between passages indicate the lack of sequencing information verifying new changes. In the case that sequencing was performed, changes in the glycoprotein (GP_1,2_) are shown or the arrow is labeled with “no changes”. Isolates recovered using reverse genetics (rEBOV) are also shown.

All experiments involving EBOV or recombinant mutants thereof were performed in a biosafety level 4 (BSL-4) containment laboratory at U.S. Army Medical Research Institute of Infectious Diseases (USAMRIID) or the Integrated Research Facility at Fort Detrick (IRF-Frederick) following approved standard operating procedures. Three reference tissue culture isolates were used in the in vitro and in vivo studies described: Ebola virus/H.sapiens-tc/COD/1995/Kikwit-9510621 clone R4415 (AIMS33146; GenBank Accession No. KT762962); EBOV/H.sapiens-tc/LBR/2014/Makona-201403621 (AIMS33895; GenBank Accession No. MW345251); EBOV/H.sapiens-tc/COD/1976/Yambuku-Mayinga (AIMS 125352; GenBank Accession No. KR063671.1). Figure 1 shows the historical records for these natural isolates.

### 2.3. Recombinant EBOV Templates

The Ebola virus/H.sapiens-wt/SLE/2014/Makona-G3864.1 genome sequence used here (EBOV/Mak-G3864.1; GenBank Accession No. KR013754.1) is identical to the basal genotype of the SL2 lineage of EBOV/Mak, which was transmitted from Sierra Leone to neighboring Liberia early on in the 2013–2016 EVD outbreak and caused the vast majority of EVD cases in Liberia [39]. The sequence of EBOV/Mak-G3864.1 was chosen for this study because it was complete (defined as the full genome sequence, including all open reading frames, flanking 3′ and 5′ untranslated regions (UTRs), and most of the leader and trailer [40]), and it originated from a patient who died of EVD. In addition to the parental EBOV/Mak-G3864.1 (r412) sequence, 3 other mutant sequences (r414, r424, and r440) were chosen for further study (Table 1).

The EBOV/H.sapiens-wt/COD/2018/Tum-BIK019 genome sequence used here (EBOV/Tum-BIK019; GenBank Accession No. MH733480) was determined using a clinical sample collected on 13 May 2018 from an EVD patient who later died in Bikoro Health District, Équateur Province, COD [8].

**Figure 2:**
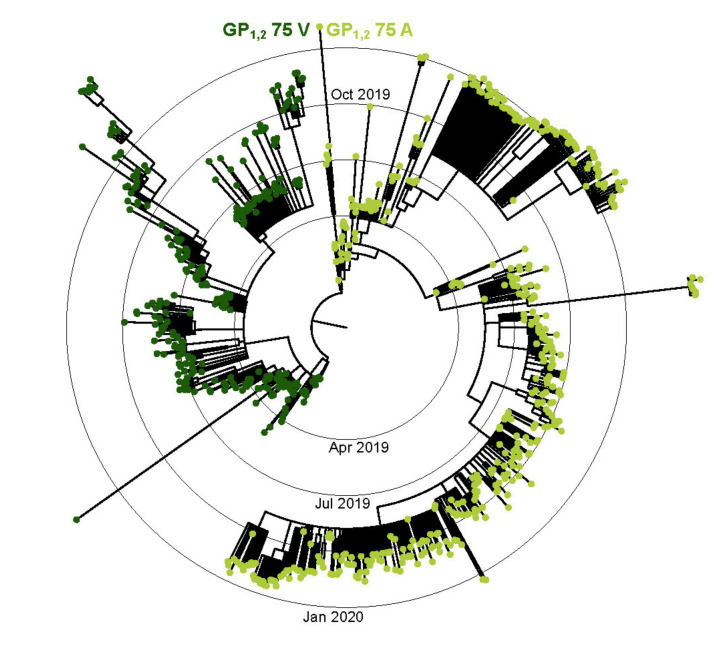
A time-scaled, maximum-likelihood inferred phylogenetic tree highlighting the valine-to-alanine change at glycoprotein (GP_1,2_) position 75. The visualization is adapted from [48]. The estimated tree is comprised of 742 EBOV/Itu sequences. Tree branches are scaled to time. Taxon tips are colored by the presence or absence of the GP_1,2_ changes.

The EBOV/H.sapiens-wt/COD/2018/Itu-18FHV089 genome sequence used here (EBOV/Itu-18FHV089; GenBank Accession No. MK007329) was determined using a clinical sample collected on 27 July 2018 from an initial EVD patient who later died in Masimbembe, Nord-Kivu Province, COD [11]. Figure 2 shows the historical records for these natural isolates.

### 2.4. Synthesis of the EBOV Genome-Encoding Plasmids and Helper Plasmids

#### 2.4.1. Genome Plasmids

The EBOV/Mak-G3864.1 (r412) genome sequence was divided into 13 pieces of approximately 1500 bp each for commercial DNA synthesis (Blue Heron Bio, Bothell, WA, USA), with the resulting plasmids designated as BB293–BB305. The fragments were designed with flanking *Sap*I sites that enabled seamless ligation to produce longer genome segments. Plasmids BB293–BB296 containing genome fragments 1–4 (nts 1–5881) were treated with restriction enzyme *Sap*I (New England Biolabs, Ipswich, MA, USA) and the resulting DNA fragments containing EBOV sub-genomic fragments were purified after agarose gel electrophoresis. The purified fragments were mixed in roughly equimolar ratios, treated with T4 DNA ligase (New England Biolabs), and amplified with flanking PCR primers. The PCR product containing joined fragments 1–4 was cloned using the Zero Blunt TOPO PCR Kit (Thermo Fisher Scientific) and sequenced (plasmid BB332). Plasmid BB336, containing pieces 5–8 (nucleotides 5882–11,975), and plasmid BB334, containing pieces 9–13 (nucleotides 11,976–18,959), were generated in the same manner, and sequencing did not reveal any additional nucleotide changes. Plasmids BB332, BB336, and BB334—each containing roughly one third of the EBOV/Mak-G3864.1 genome—were digested with *Sap*I and *Not*I, ligated and then ligated into pJazz OK (Lucigen, Middleton, WI, USA). The pJAZZ plasmid containing the full EBOV/Mak-G3864.1 genome was sequence-confirmed and designated BB341. The entire viral genome was transferred by Gibson assembly into a new plasmid backbone between a 5′ T7 RNA polymerase promoter and a 3′ hepatitis D virus ribozyme (HDZ) T7 RNA polymerase transcription terminator (T7_term_) sequence (plasmid BB412 encoding recombinant rEBOV/Mak-G3864.1 mutant r412). rEBOV/Mak-G3864.1 mutants r414, r424, and r440 (Table 1) were created by introducing the desired mutations into PCR primers that were then used to amplify sub-genomic segments from plasmid BB412. The sub-genomic fragments with the desired mutations were then transferred by Gibson assembly into the full genome plasmids BB414, BB424, and BB440, respectively.

DNA versions of the EBOV/Itu-18FHV089 and EBOV/Tum-BIK019 genomes were commercially synthesized as approximately 30 fragments with average lengths of approximately 500 bp (Twist Bioscience, San Francisco, CA, USA). Each fragment had 50-bp overlaps with the respective upstream and downstream fragments. Sequence-verified individual short fragments were PCR-amplified with Phusion High-Fidelity Polymerase (Thermo Fisher Scientific) and pooled in groups of 5 or 6 for Gibson assembly. Products of subgenomic Gibson assembly reactions were reamplified by PCR to generate 2.5- to 3-kbp fragments that were subsequently joined by a second Gibson assembly to generate complete genome clones (plasmids BB865 [encoding rEBOV/Itu-18FHV089] and BB866 [encoding rEBOV/Tum-BIK019]).

#### 2.4.2. Helper Plasmids

The sequences encoding EBOV/Mak-G3864.1 L, NP, VP35, and VP30 were codon-optimized for increased expression in human cells (Thermo Fisher Scientific). The codon-optimized open reading frames were cloned into the cytomegalovirus (CMV) promoter-driven expression vector pWRG-7077 [49] by Gibson assembly to generate plasmids BB401, BB402, BB406, and BB405.

#### 2.4.3. Plasmid Sequencing

All genome and helper plasmids were sequenced on a PacBio RSII System (Pacific Biosciences, Menlo Park, CA, USA) to confirm sequence identities with chosen GenBank sequences. Full genome plasmid sequences have been deposited in GenBank with accession numbers MN975530–MN975535, and MW308592–MW308595. Sequencing revealed a silent C3579A change in the *VP35* gene in plasmid BB412 (Table 1). This change fortuitously disrupted an *Ahd*I restriction site, and the change was therefore left as a tag to allow for distinguishing recombinant virus from the natural isolate.

### 2.5. EBOV/Mak-G3864.1 Minigenome

The EBOV/Mak-G3864.1 sequence r412 genome leader and trailer and plasmid backbone were PCR-amplified from plasmid BB412. Enhanced green fluorescent protein (eGFP) was amplified from plasmid pWRG7077-eGFP [49] with primers containing short overlaps to the leader and trailer regions. The 2 PCR products were joined by Gibson assembly to create an EBOV/Mak-G3864.1 minigenome plasmid (plasmid BB427). To examine eGFP expression from the minigenome, RNA was produced from the minigenome plasmid by in vitro transcription using the HiScribe T7 High Yield RNA Synthesis Kit (New England Biolabs). On Day 0, HEK 293T cells were plated in 6-well dishes to achieve approximately 50% confluency the following day. The next day (Day 1), cells of each well were transfected using TransIT-2020 Transfection Reagent (Mirus Bio, Madison, WI, USA) with a mixture of 650 ng BB402, 330 ng BB406, 200 ng BB405, and 1.3 μg BB401 helper plasmids. On Day 2, wells were transfected with 2.5 μg of minigenome RNA. eGFP expression was examined by fluorescent microscopy 1 d after RNA transfection.

### 2.6. Recombinant EBOV Rescue

EBOV antigenomic RNAs were produced by in vitro transcription of the appropriate full-genome plasmid (BB412, BB414, BB424, BB440, BB865, and BB866) using the HiScribe T7 High Yield RNA Synthesis Kit (New England Biolabs). In vitro transcribed RNA was purified through lithium chloride precipitation and ice-cold 70% ethanol. On Day 0, HEK 293T cells were plated in a BSL-2 laboratory in 6-well dishes to achieve approximately 50% confluency on Day 1. On that day, cells of each well were transfected using TransIT-2020 Transfection Reagent (Mirus Bio) with a mixture of 650 ng BB402, 330 ng BB406, 200 ng BB405, and 1.3 μg BB401 helper plasmids. For negative-control wells, BB405 was replaced with a plasmid-only expressing eGFP, pWRG7077-eGFP. On Day 2, plates were transferred to a BSL-4 laboratory, where 2.5 μg of the appropriate EBOV genome RNA was then transfected per well using the TransIT-mRNA Transfection Kit (Mirus Bio). Seven days after RNA transfection, half of the supernatant volume from a well with transfected cells was transferred to fresh Huh7 cells (Passage 1), and the other half was retained at −80 °C in case cell passages needed to be repeated. Passage 2 was performed in the same manner 7 d after Passage 1. Seven days after Passage 2, cells were evaluated for visible cytopathic effect (CPE) and, if no CPE was apparent, the supernatant of that well was transferred again onto fresh cells.

Once a CPE-positive culture was obtained, supernatant was homogenized in TRIzol-LS (Thermo Fisher Scientific) and removed from containment for sequence analysis as described above. For additional confirmation, rescued virus was used to infect fresh Huh7 cells, incubated for 7 d, and fixed in 10% buffered formalin for removal from the containment suite. After removal from the containment suite, formalin was removed from the cells, and the fixed cells were rinsed with phosphate-buffered saline (PBS) to remove residual formalin. The cells were permeabilized with cold methanol, and after a short incubation, the methanol was removed, and the cells were rinsed with PBS to remove residual methanol. After blocking with a solution consisting of 3% bovine serum albumin (BSA) in PBS, the cells were incubated with an anti-EBOV glycoprotein (GP_2_) monoclonal antibody (AdiMab, Lebanon, NH, USA; Cat. No. 15742), rinsed with PBS, and then incubated with a goat anti-human immunoglobulin G (IgG) secondary antibody labeled with Dylight 488 (AbCam, Cambridge, MA, USA; Cat. No. ab96907) to allow for detection by fluorescent microscopy.

### 2.7. Small Molecules

Compounds 3.47 [50], 1412 (3–16), 1413 (3–17), 1466 (3–18), and 1476 (3–21) were synthesized as previously described [51].

### 2.8. EBOV Assay in HeLa Cells

For half-maximal effective concentration (EC_50_) and cytotoxic concentration (CC_50_) determination, dose responses were conducted in BSL-4 at USAMRIID. HeLa cells were seeded at 2000 cells per well in 384-well plates (Aurora Black Square ULB 384 IQ-EB, 188-μm Clear Film Bottom; Cat. No. 1052-11130-S) 1 d prior to virus inoculation. The HP-D300 digital (Hewlett Packard) dispenser was used to generate an 8-point dose response with a 3-fold step dilution starting at 10 (3.47 and 1476) or 20 (1412, 1413, and 1466) μM. Each dose was dispensed in 4 replicates. For each 384-well plate, 1 compound was used for quality control. Dimethyl sulfoxide (DMSO) concentration in all wells was normalized 1%. Two hours after treatment, cells were inoculated with different EBOV variants and mutants: EBOV/Mak-201403261 (multiplicity of infection [MOI] = 1.5), EBOV/Mak-r412 (MOI = 0.1), EBOV/May-tc (MOI = 1), or EBOV/Mak-r440 (MOI = 8). Cells were fixed in 10% formalin 48 h after virus inoculation or at least 48 h before immunostaining. MOIs for virus inoculations were selected based on optimization assays that produced a ≈50–90% infection rate on HeLa cells at the assay endpoint. Titers of virus stocks were determined via plaque assays on Vero cells. The different MOIs of EBOV variants used for HeLa cells infections might reflect the variants’ differing efficiencies in infecting HeLa cells.

### 2.9. Immunostaining

Detection and quantification of viral infection in assay plates was performed as described previously [52] using a high-content imaging (HCI) assay to measure viral antigen production after immunofluorescent labelling. To detect viral infection, inactivated plates were transferred into a BSL-2 lab for immunostaining. Assay wells were incubated for 1 h with permeabilization/blocking buffer containing 3% BSA, 0.1% Triton, and PBS. Assay wells were then stained for 1 h with a primary antibody against EBOV GP (mm 6D8), diluted 1000-fold in blocking buffer. Following incubation, the primary antibody was removed, and the cells were washed 3 times with 1× PBS. Cells were subsequently incubated for 1 h with DyLight-488-conjugated goat anti-mouse IgG (Thermo Fisher; Cat. No. 35502B), diluted 1000-fold in blocking buffer. Cells were also stained with Hoechst3332 (Thermo Fisher) for nuclei detection and CellMask Deep Red (Thermo Fisher; Cat. No. C10046) for optimal detection of cytoplasm for at least 30 min before image acquisition.

### 2.10. Image and Data Analysis

Images were acquired on the PerkinElmer Opera confocal imaging instrument using 10× Air objective. (Five fields were acquired per well.) Signal from virus staining was detected by a charged-coupled device (CCD) camera at a 488 nm emission wavelength, nuclei staining at 400 nm, and cytoplasm staining at 640 nm. Image analysis was performed simultaneously with image acquisition using PerkinElmer Acapella algorithms. The assay quality of each plate was assessed using the Z prime factor (Z′). Assay results were considered acceptable if Z′ was greater than 0.5. Additionally, the percent infection rate, consistency of cell counts per well, and results for internal positive control compounds were also used as quality-control criteria for each plate. Dose response curve analysis (to determine EC_50_ values) was performed using GeneData screener software or GraphPad Prism 9.1.2 applying Levenberg–Marquardt algorithm (LMA) for curve-fitting strategy. Curve-fittings were done using 2-parameter non-linear regression. One-way ANOVA analyses were conducted using GraphPad Prism 9.1.2.

### 2.11. Sequencing Data Analysis

Illumina deep sequencing data generated from the rescued virus samples was analyzed with an in-house software pipeline, VSALIGN [53], to determine consensus changes and the frequencies of minor variant populations.

### 2.12. Laboratory Mouse Experiments

IFNAR^−/−^ laboratory mice (B6.129S2-Ifnar1tm1Agt/Mmjax; C57BL/6 background), were purchased from Jackson Laboratories (Bar Harbor, ME, USA). The mice were housed in microisolater cages and provided chow and water ad libitum. Mice were exposed intraperitoneally to approximately 1000 PFU of EBOV/Mak-TD, EBOV/Kik, rEBOV/Itu, rEBOV/Tum, rEBOV/Mak-G3864.1, or a mutant of rEBOV/Mak-G3864.1. At the time of exposure, mice ages ranged from 11 to 27 weeks. Mice were housed by sex and age in groups of 10. Mice were observed daily for clinical signs of disease, including but not limited to reduced grooming, hypoactivity, and weight loss, or lethality. Observations were increased to a minimum of twice daily while mice were exhibiting signs of disease. Moribund mice were humanely euthanized based on criteria approved by USAMRIID’s Institutional Animal Care and Use Committee.

## 3. Results

### 3.1. Recombinant EBOV Rescue

#### 3.1.1. Selection of EBOV Isolate Sequences for De Novo Genome Synthesis and Rescue

We chose to synthesize genomes of isolates representing three EBOV variants from recent EVD outbreaks. Complete or near-complete genome sequences for EBOV/Mak, EBOV/Tum, and EBOV/Itu were available in GenBank but, relatively few, if any, natural isolates were available for laboratory study. For each variant, we chose coding-complete genome sequences (containing sequence data on all open reading frames) that had been derived from clinical isolates of lethal EVD cases (EBOV/Mak-G3864.1, EBOV/Tum-BIK019, and EBOV/Itu-18FHV089).

#### 3.1.2. Plasmid Establishment and Sequence Verification

The genomes of each of the selected isolates were commercially synthesized in multiple fragments and then assembled into full genome clones. Publicly available sequences contained large parts of the genomic 3′ leader and 5′ trailer (untranscribed regions flanking the open reading frames that are important for genome replication, transcription, and packaging) but were missing 1–15 terminal residues from both ends. The 3′ and 5′ extremes of the leader and trailer are highly conserved in EBOV, so the missing ends from each isolate were filled in using the conserved sequences to generate complete genomes.

The parental EBOV/Mak-G3864.1 clone (designated “r412”) was used to generate three additional mutants of interest of EBOV/Mak (Table 1). Clone r414 incorporated a silent mutation in the *NP* gene, a nonsense mutation that resulted in truncation of the last seven amino-acid residues of VP30, and a change in the *VP24* gene UTRs [41]. Clone r424 incorporated a double mutation in the *NP* gene UTRs that has been shown to affect NP expression in an EBOV minigenome assay [42]. Clone r440 incorporated a single nucleotide change, which resulted in a valine-to-alanine residue substitution in the EBOV GP_1,2_ A82V. Virus sequences from the very beginning of the 2013–2016 EVD epidemic encoded 82A, but the 82V genotype emerged early and represented greater than 90% of the all genomes sequenced during the epidemic [39]. The A82V change is known to enhance EBOV infection in nonhuman primate cells and to decrease infectivity in bat-derived cell lines [26,27,28,43,44,45,46], but its effect on in vivo infections remains to be determined [47].

In addition to the designed changes, it was discovered after sequence confirmation that clone r412 had picked up a silent mutation in the *VP35* gene. This mutation abrogated an *Ahd*I restriction enzyme site and therefore was kept for use as a marker to distinguish recombinant virus from natural isolates, if needed. Clone r424 and clone r440 (derived from r412) also had this silent mutation, but clone r414 had reverted to the wild-type *VP35* sequence.

Finally, plasmids containing the full genomes of EBOV/Itu and EBOV/Tum were designated r865 and r866, respectively (Table 1).

Most filovirus minigenome and reverse-genetics systems involve multi-plasmid transfections of four helper plasmids (encoding filovirus structural proteins NP, VP35, VP30, and L under enterobacteria phage T7 DNA-directed RNA polymerase promoter (T7 RNA polymerase control), a plasmid expressing T7 RNA polymerase, and a plasmid from which a filovirus RNA minigenome or a full filovirus RNA genome or antigenome can be transcribed [37]. Using the EBOV/Mak-G3864.1 sequence, we generated helper plasmids containing the *NP*, *VP35*, *VP30*, and *L* open reading frames that were codon-optimized for increased expression in mammalian cells. We also generated an EBOV/Mak-G3864.1 sequence r412-based minigenome plasmid encoding a reporter gene, eGFP, flanked by the EBOV/Mak-G3864.1 leader and trailer regions. EBOV minigenome systems can be used to study viral genome replication and transcription outside of BSL-4 containment, as the minigenome lacks several or all of the viral genes and does not produce replication-competent viruses. Reporter expression (eGFP in this study) can be detected when the minigenome is replicated and transcribed by helper proteins provided in trans [37].

### 3.2. EBOV Rescue

**Figure 3:**
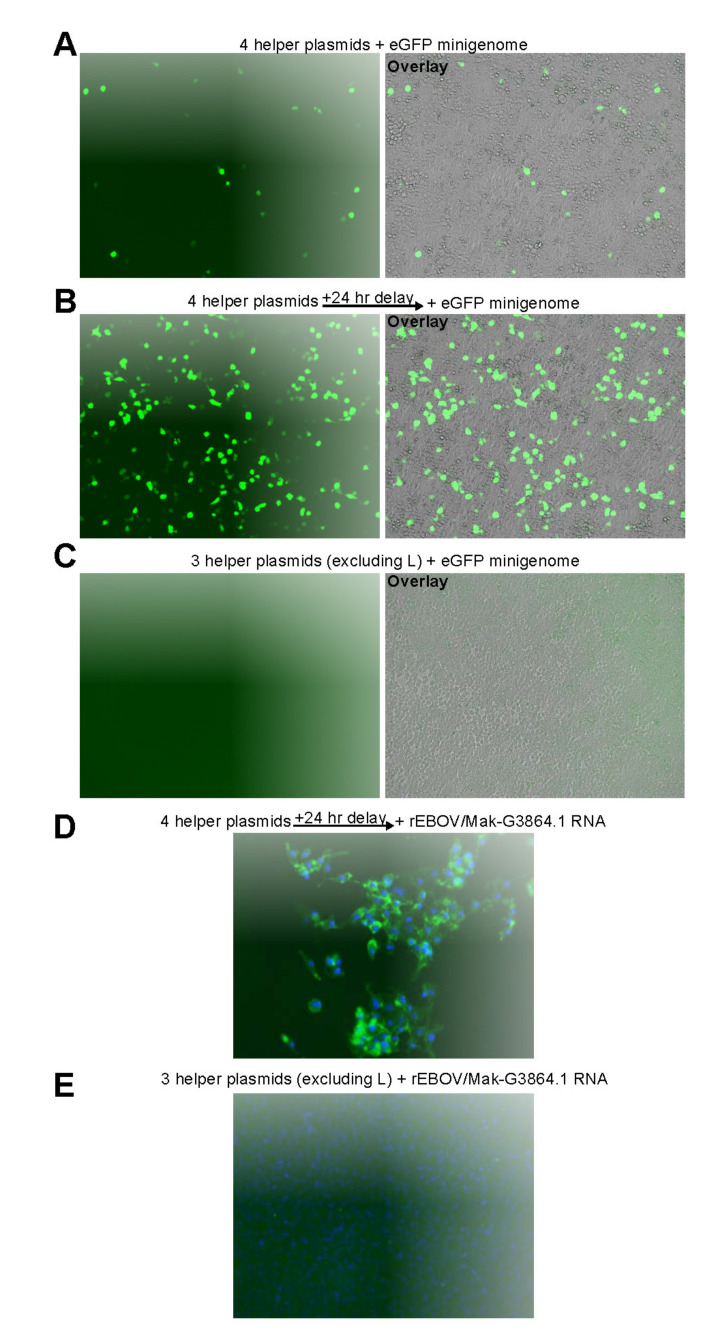
EBOV/Mak-G3864.1 minigenome and virus rescues. HEK 293T cells were transfected with expression plasmids having codon-optimized open reading frames encoding the EBOV/Mak-G3864.1 helper proteins NP, VP35, VP30, and L. eGFP expression was examined 48 h after helper plasmid transfections. (**A**) In addition to helper protein-encoding plasmids, a plasmid expressing T7 RNA polymerase and a minigenome plasmid encoding eGFP, flanked by the EBOV/Mak-G3864.1 leader and trailer sequences, were transfected simultaneously. (**B**) 24 h after transfection of helper plasmids, minigenome RNA (generated by in vitro transcription) was transfected. (**C**) Similar to (**B**), but the L-encoding plasmid was excluded from the helper plasmid mix. (**D**) HEK 293T cells were transfected with all four helper plasmids or (**E**) with a mix excluding the L-encoding plasmid. 24 h later, rEBOV/Mak-G3864.1 antigenomic RNA was transfected into the same cells. Transfection supernatants were passaged onto fresh Huh7 cells 7 d post-transfection (Passage 1), and Passage 2 was initiated in fresh Huh7 cells 7 d after Passage 1. Passage 2 supernatants were transferred onto fresh Huh7 cells (Passage 3), incubated for 4 d, and then fixed for immunofluorescence. Expressed EBOV GP_2_ was detected with an anti-EBOV GP_2_ monoclonal antibody (green). Cell nuclei were stained with DAPI (blue).

As described for other EBOV minigenome systems [36,37], transfection of the EBOV/Mak-G3864.1 sequence r412-based minigenome plasmid together with the four helper plasmids in trans resulted in eGFP expression (Figure 3A). Attempting to increase minigenome expression efficiency, we repeated the experiment but, instead of minigenome plasmid transfection, cells were transfected initially with the helper plasmids, followed 24 h later by transfection of minigenome plasmid-derived in vitro-transcribed minigenome RNA. Compared to an all-plasmid transfection, delayed transfection of the minigenome RNA resulted in an approximately 10-fold increase in eGFP-positive cells (Figure 3B). Replication and transcription of the minigenome requires expression of all four helper proteins. Consequently, excluding the L helper plasmid from the transfections abrogated expression of eGFP as expected (Figure 3C). Exclusion of NP, VP35, or VP30 also abrogated expression of eGFP from the minigenome (data not shown).

Due to this noticeable increase in minigenome rescue efficiency, we decided to follow the same RNA transfection protocol for EBOV rescues. Virus rescues were performed using the four EBOV/Mak-G3864.1 helper plasmids and full antigenome RNAs transcribed from full-length recombinant EBOV (rEBOV) genome-containing plasmids. Human embryonic kidney (HEK) 293T cells were transfected with the helper plasmids and were transferred to BSL-4 containment. The same cells were transfected with full antigenomic RNAs 24 h after helper plasmid transfection. Supernatants from the transfected HEK 293T cells were passaged on human Huh7 cells for either one or two additional passages until cytopathic effect became apparent. Supernatants from wells with CPE were passaged onto fresh Huh7 cells, incubated for several days, fixed, and stained with an antibody to detect EBOV GP_1,2_ expression by immunofluorescence. As shown in Figure 3D, this approach resulted in efficient rescue of rEBOV/Mak-G3864.1 mutant r412. The genomes of rescued viruses were sequenced on an Illumina MiSeq to confirm that no changes had been introduced during rescue. After the rescue protocol was established with r412, it was used to successfully rescue rEBOV/Mak-G3864.1 mutants r414, r424, r440, r865, and r866—again without rescue-induced sequence changes.

The EBOV genome is known to acquire cell-culture-adaptive mutations during serial passage. The RNA editing site in the EBOV *GP* gene is a stretch of seven uridylyls (a 7U sequence) that changes to an 8U sequence during serial passage in Vero cells [29,31]. The 7U sequence found in clinical samples of EVD patients and early cell-culture passages of EBOV resulted in the expression and secretion of a protein of unknown function, sGP. Co-transcriptional frameshifting caused by the addition of the extra uridylyl to nascent *GP* mRNAs resulted in expression of virion GP_1,2_ [29,31]. Conversely, the 8U sequence found in genomes of passaged EBOV led to primary expression of GP_1,2_, and secondary expression of sGP via co-transcriptional editing, with the sGP-to-GP_1,2_ expression levels reversing. The selective pressure driving conversion of 7U to 8U genomes during EBOV cell-culture passage is unknown, but this pressure appears to be reversed in vivo as 8U EBOV quickly reverts to 7U after animal inoculation [29,31,32]. Another well-studied example of EBOV cell-culture adaptation is the GP_1,2_ T544I change that occurs within a small number of passages of EBOV on some nonhuman primate cell lines [30]. T544 is located within the internal fusion loop of GP_2_, and conversion of T544 to I544 potentiates viral entry into nonhuman-primate-derived cell lines [44].

To determine how quickly cell-culture adaptation occurs in recombinant EBOVs, the rEBOV/Mak-G3864.1 mutant r412 reverse-genetic system was used to rescue virus in human HeLa, Huh7, and Huh7D-12/T7 cells (Huh7 cells constitutively expressing hepatitis delta antigen and T7 RNA polymerase) and grivet (*Chlorocebus aethiops*) Vero E6 cells using a total of eight different conditions (Table 2). The rescued viruses were subjected to deep sequencing to identify any consensus changes (appearing in >50% of the population) or minor variant populations (5–50%) As shown in Table 2, only one of the recombinant virus stocks (rescued from Vero E6 cells) was associated with consensus genome changes, resulting in single amino-acid residue changes each in VP40 and VP30. Minor variants were detected under four of the eight conditions tested, ranging in frequency from 7.9–38.9% of the virus population. 8U genomes were detected at the minor variant level in seven samples (maximally 2.4% of the population) but accounted for 9.1% of the population in one sample. Importantly, the T544I change was not detected in any of the datasets, indicating that limited passaging (no more than two passages) of rescued EBOVs prevents extensive cell-culture adaptation [30].

#### 3.2.1. Cell Culture Adaptations Can Alter Susceptibility to MCMs

Compound 3.47 is a small molecule inhibitor of EBOV infection that targets the host receptor, NPC intracellular cholesterol transporter 1 (NPC1) [49,53,54]. Murine leukemia virus particles pseudotyped with EBOV/Mak GP_1,2_ are more sensitive to inhibition by 3.47 than particles pseudotyped with EBOV/Mak GP_1,2_ A82V or 1976 EBOV variant Yambuku (EBOV/Yam) variant GP_1,2_ [46]. The antiviral action of 3.47 was mapped outside of the mucin-like domain (MLD), since pseudotype mutants bearing a deletion of the MLD (GP_1,2_ residues 309–489) were equally inhibited by 3.47. Modification of the EBOV/Yam GP_1,2_ at position 544 (T544I) had a similar effect [46]. T544I was previously demonstrated to be an artificial product of EBOV adaptation to Vero E6 cells in both the EBOV/Mak and 1995 EBOV variant Kikwit (EBOV/Kik) background [30,54].

**Figure 4:**
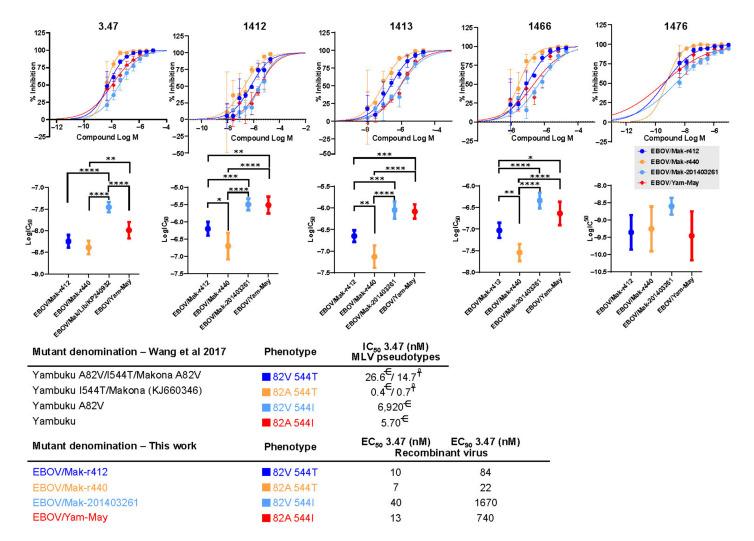
Antiviral activity of the entry inhibitor 3.47 and its analog. Dose response studies were performed on HeLa cells that were pre-treated with increasing concentrations of the indicated compounds for 2 h and subsequently inoculated with the indicated EBOV variant (EBOV/Mak-r412, EBOV/Mak-r440, EBOV/Mak-201403261, or EBOV/Yam-May). Cells were fixed in formalin 48 h after virus inoculation and immunostained for high-content quantitative image-based analysis with EBOV-specific antibodies. Infection rates were normalized to DMSO-treated samples. mean  ±  s.d. from four replicates are shown. Mean EC_50_ values with 95% confidence interval (CI) of each dose response are shown. One-way ANOVA (Tukey’s multiple comparisons test; *n* = 32) was used to calculate a *p* value [0.1234 (ns), 0.0332 (*), 0.0021 (**), 0.0002 (***), <0.0001 (****)]. In the cited experiments, murine leukaemia virus (MLV) was pseudotyped with glycoproteins (GP_1,2_s) of EBOV Yambuku-Mayinga ^€^ or Makona ^¥^ containing the indicated residues.

To evaluate the effect of GP_1,2_ variants A82V and T544I on susceptibility to the entry inhibitor 3.47 and its analogs in the context of replication-competent viruses, we performed dose response studies with two of our recombinant EBOV isolates and two natural EBOV isolates (Figure 4 Recombinant isolates EBOV r412 and r440 differ only at GP_1,2_ residue 82 (with r412 encoding a valine residue at that position) and r440 (encoding an alanine residue). Both of these isolates encode a threonine residue at position 544, reflecting the genotype seen in clinical isolates. Natural isolates EBOV/Mak-201403261 (GenBank accession number KP240932) and tissue-culture adapted EBOV/Yam-May (EBOV/May-tc) both encode an isoleucine at position 544. The change from T544 to I544 during passage in Vero E6 cells was documented for EBOV/Mak-201403261. The original residue for EBOV/Yam-May is not known, as there is no genome sequence available for the unpassaged (P0) clinical isolate of this virus. These two natural isolates differ at position 82, with either a valine (EBOV/Mak-201403261) or alanine (EBOV/Yam-May) residue encoded at that position. Outside of the MLD region, the EBOV/Mak and EBOV/Yam variants only differ at two amino-acid positions: position 498 (V in EBOV/Mak, A in EBOV/Yam) and 262 (A in EBOV/Mak, T in EBOV/Yam). Together, the four viruses represent all possible combinations of GP82A or GP82V with GP544T or GP544I.

The potency (IC_50_) results of compound 3.47 and its analogs—1412, 1413, 1466, and 1476—demonstrate significant differences in sensitivity of the virus variants to the compounds (Figure 4 and Table 3). For all compounds tested, potency was highest against EBOV/Mak-r440 (82A/544T) and lowest against EBOV/Mak-201403261 (82V/544I). IC_50_ differences ranged from six-fold (3.47) to 19-fold (1412) (Table 3). Comparison of results for variants EBOV/Mak-201403261 (82V/544I) and EBOV/Mak-r412 (82V/544T) suggest that potency of the compounds was reduced by three- to four-fold due to the T544I variation on the 82V background (Table 3). On the 82A background, the T544I variation resulted in a two- to 17-fold reduction in potency (EBOV/May-tc versus EBOV/Mak-r440). The A82V variation had more significant effect on sensitivity to the compounds on the 544T background (1.4- to 5-fold reduction in IC_50_) than on the 544I background (1.1- to 2-fold reduction in IC_50_).

Overall, these results suggest that virus modifications associated with cell-culture adaptations (such as T544I) might result in virus mutants with different susceptibilities to MCMs compared to the clinical isolates from which they are derived.

#### 3.2.2. Recombinant EBOV/Mak-G3864.1 Mutants Cause Weight Loss in Interferon-Alpha/Beta Receptor Knockout (INFAR^−/−^) Laboratory Mice

**Figure 5:**
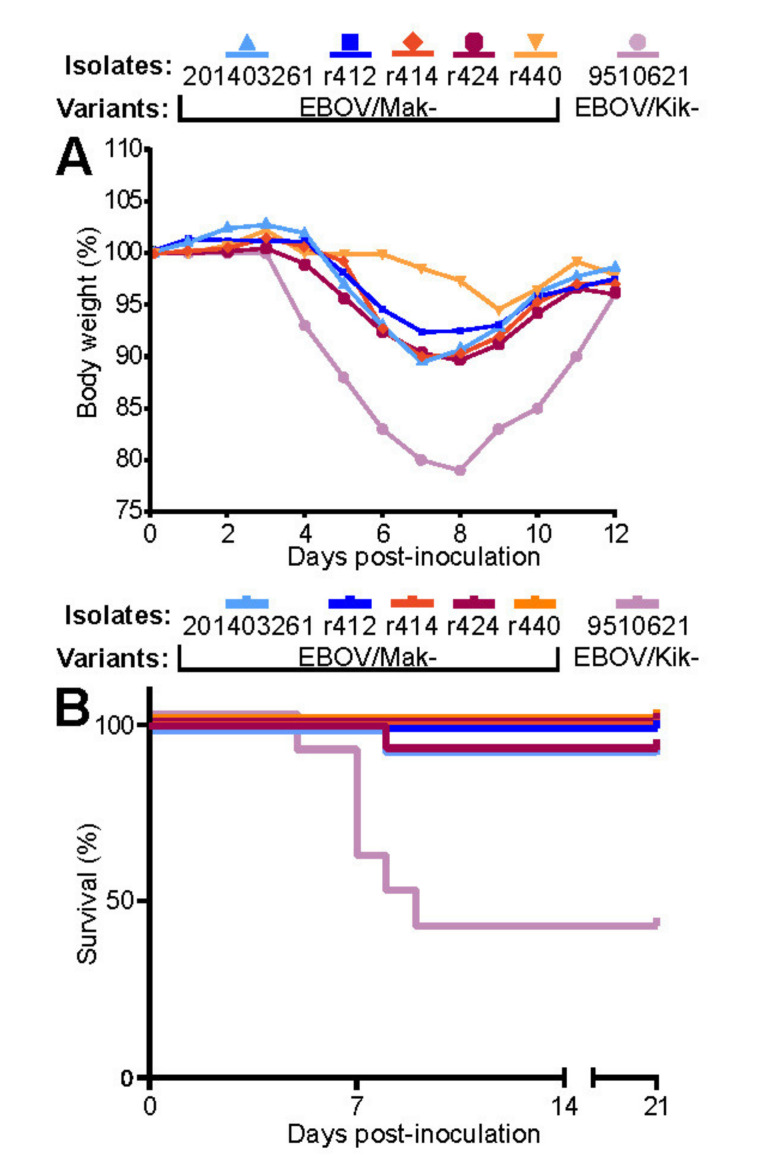
Recombinant EBOV/Mak-G3864.1 caused weight loss in INFAR^−/−^ mice. Groups of 10 INFAR^−/−^ laboratory mice were inoculated intraperitoneally with ≈1000 PFU of rEBOV/Mak-G3864.1 mutant isolates r412, r414, r424, or r440 or natural isolates EBOV/Mak-201403261 (TD) or EBOV/Kik-9510621 [10]. Weight (**A**) and survival (**B**) were monitored daily for up to 21 d. INFAR^−/−^ = interferon-alpha/beta receptor knockout.

**Figure 6:**
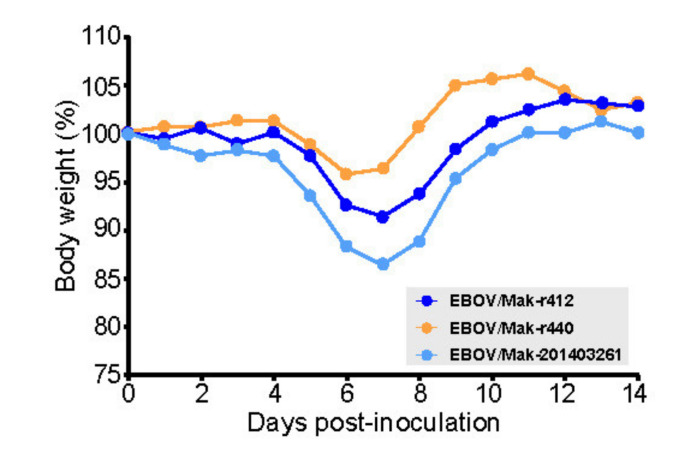
Recombinant EBOV/Mak-G3864.1 mutant r412 caused increased weight loss than r440 in INFAR^−/−^ mice. INFAR^−/−^ laboratory mice were infected with ≈1000 PFU of rEBOV/Mak-G3864.1 mutants r412 (GP_1,2_ 82V) or r440 (GP_1,2_ 82A) or the natural isolate EBOV Mak-201403261. Weights were monitored daily for 14 d. INFAR^−/−^ = interferon-alpha/beta receptor knockout.

In the IFNAR^−/−^ laboratory mouse model of EVD (which, in contrast to non-immunosuppressed laboratory mouse models [55], does not require rodent-adapted EBOV), intraperitoneal injection of wild-type EBOV leads to significant weight loss at 5–8 d after infection [56]. To evaluate pathogenicity and virulence of recombinant EBOVs in this model, groups of 10 laboratory IFNAR^−/−^ mice were inoculated with 1000 PFU of rEBOV/Mak-G3864.1 mutants r412, r414, r424, or r440. Natural (non-recombinant) EBOV/Mak-201403261 was used as a control to clinically compare recombinant isolates to a natural isolate. A natural (non-recombinant) isolate of EBOV/Kik, EBOV/Kik-9510621, obtained during an EVD outbreak in Zaire in 1995, was included as a second control, as this virus is known to cause death in the IFNAR^−/−^ laboratory mouse model [56]. The overall weight loss caused by rEBOV/Mak-G3864.1 mutants r412, r414 and r424, in IFNAR^−/−^ laboratory mice was similar in timing and magnitude to the weight loss caused by EBOV/Mak-201403261, However, the pattern of body weight loss of r440 appears to be even milder, which is interesting considering that this variant was the one more sensitive to the compounds tested here (Figure 5 and Figure 6), indicating that the reverse-genetics-derived viruses were equally as pathogenic and virulent as the natural isolate in this model. Lethality was equally similar: Single deaths occurred in both groups. As expected, mice inoculated with EBOV/Kik-9510621 developed severe weight loss and were found dead (Figure 5A,B).

In a separate experiment, we compared rEBOV/Mak-G3864.1 mutants r412 and r440, which differ at a single nucleotide position (nt 6283, resulting in either a valine [mutant r412] or alanine [mutant r440] residue at GP_1,2_ position 82). Mice infected with either mutant began to lose weight around Day 4, but those infected with mutant r440 lost slightly less weight than those infected with mutant r412 (Figure 6). Both mutants caused milder weight loss than EBOV/Mak-201403261.

## 4. Discussion

New sequencing technologies have dramatically shortened the time required to obtain complete virus genome sequence information during an outbreak. High-throughput sequencing technologies can be used to obtain near-complete viral genome sequences in less than one day [23] and complete genome sequences in a couple of days [21]. Although genome sequence information can be rapidly obtained, dissemination of virus stock material still presents a bottleneck for initiating MCM development.

We developed several fully synthetic EBOV reverse-genetics systems starting solely from genome sequence information. Virus genomes were commercially synthesized in multiple fragments that were then assembled into complete genomes. Our EBOV/Mak parental clone, rEBOV/Mak-G3864.1 genotype r412, represents an isolate identical to the basal genotype of the SL2 lineage responsible for the majority of EVD cases in Liberia during the 2013–2016 outbreak. Mutant r412 encodes a valine at GP82, reflecting a change that occurred early in the outbreak and was seen in the vast majority of sequenced EBOV/Mak genomes. 

Three additional mutants were constructed from this parental clone, reflecting genotypes of interest that had been detected during that outbreak. Mutant r414 truncated the last seven amino acids of the *VP30* gene, which has been shown to increase expression from an EBOV minigenome assay [57]. Mutant r424 harbored two nucleotide changes in the *VP24* gene UTRs that have also been shown to alter expression in a minigenome assay. Mutant r440 differs from r412 by a single amino acid at GP_1,2_ position 82, encoding an alanine residue at this position as opposed to the valine residue in r412.

Contrary to classic filovirus reverse-genetics protocols, we rescued EBOVs by providing EBOV antigenomic RNAs 24 h after helper plasmid transfection instead of simultaneous transfection of helper plasmids and an EBOV genome-containing DNA plasmid. This protocol is advantageous as it simplified EBOV rescues, which have to be performed under BSL-4 containment. Under the modified protocol, the helper plasmids are transfected in a BSL-2 laboratory. The following day, the helper-plasmid-transfected cells and genomic RNA are kept separate and transported to BSL-4 containment, reducing the handling time in BSL-4. The RNA is then combined with transfection reagent and added to cells after a 3- to 5 min incubation. This protocol therefore minimizes time and liquid manipulations in BSL-4 containment.

One important consideration during MCM development is that the virus stocks used should be as similar to clinical isolates as possible to avoid spurious assay results. Cell-culture passaging of EBOV is known to drive specific adaptations, some of which alter virus replication kinetics and/or response to candidate MCMs [29,32]. We investigated the speed at which cell-culture adaptations arise in recombinant EBOV preparations by rescuing and propagating virus stocks in several different cell lines. Under one condition, during propagation in human HeLa cells, we detected a cell-culture adaptation in GP_2_ that is known to increase the infectivity of vesicular stomatitis Indiana virus (VSIV) particles bearing EBOV GP_1,2_ (a frequently used BSL-2 surrogate model for EBOV infection). Passaging of VSIV-EBOV GP_1,2_ in grivet Vero E6 cells resulted in a spontaneous mutation leading to a D552N change, which appeared in approximately 8–9% of viruses after one or two passages [54]. This same cell-culture adaptation was present in approximately 30% of our rescued EBOV population after two rounds of propagation in HeLa cells. Importantly, D552N is the only known cell-culture adaptation that we identified under the eight tested rescue conditions. Additionally, we did not detect another common cell-culture adaptation in GP_2_ T544I under any conditions. 

Another marker of cell-culture adaptation in EBOV is the switch in the *GP* RNA editing site from majority-7U to majority-8U. The fraction of rescued virus genomes bearing 8U never rose above 10% in our rescue attempts, with the majority of rescues hovering around 1–2% 8U. The low frequency of detection of cell-culture adaptations found after EBOV rescue and limited cell-culture passaging suggest that EBOV stocks rescued by reverse genetics may be ideal starting points for candidate MCM efficacy testing, as such stocks more closely resemble clinical virus sequences than extensively passaged natural isolates. Additionally, when EBOV was rescued and propagated in human Huh7 cells, we did not detect any mutations in the virus population at greater than 5%, indicating that Huh7 may be an ideal cell line for rescuing virus stocks identical to clinical isolates, as previously reported [58].

All of the EBOV/Mak mutants were successfully rescued and examined for pathogenicity in a laboratory mouse model of EVD. Although the mutations included in r414 and r424 affect reporter expression in minigenome assays, EBOV/Mak mutants r414 and r424 resulted in a similar phenotype of infection to that caused by parental r412 in the mouse model. Additionally, all of the mutants caused similar amounts of weight loss compared to a natural isolate of EBOV/Mak, providing evidence that viruses derived by reverse genetics behave similarly to natural isolates. These findings also indicate that the GP_1,2_ A82V does not substantially alter pathogenicity in this model. Mice infected with mutant r440 lost slightly less weight than those infected with mutant r412, but additional experiments are needed to determine the significance of this result. Our results were similar to those reported in a previous study that leveraged nine different natural EBOV/Mak isolates (three Guinean [C05, C07, and C15], one Malian [29], and five Liberian [304, 1225, 2596, 3697, and 4156 isolates] in the IFNAR^−/−^ mouse model [47]. In those experiments, minimal differences in pathogenicity were observed between isolates obtained early in the 2013–2016 EVD outbreak (bearing GP82A, as in r440) and those obtained later in the outbreak (bearing GP82V, as in r412). However, a statistically significant difference in weight loss was measured between the two groups, with the early isolates causing more weight loss than the later isolates [47]. This observation is the opposite of what we saw in our study, in which we measured increased weight loss in mice inoculated with mutant r412, which mimics the later isolates. Whereas our r412 and r440 mutant viruses differed at a single nucleotide (and amino acid) position, Marzi et al. used natural virus isolates that differed not only at GP_1,2_ position 82 but also had additional changes, including L D759G and NP R111C [47], both of which impact viral fitness [43,59]. It is possible that weight loss caused by changes in GP_1,2_ position 82 is context-dependent, which could explain the different observations between our study and that of Marzi et al. This observed difference also highlights one benefit of using viruses generated by reverse genetics: Precise manipulation of reverse-genetics clones can allow highly controlled comparisons of viruses differing by as little as a single nucleotide change.

The phenotypes involving variants at positions 82 and 544 in GP have been significantly studied in the last few years with the use of pseudotyped viral vectors [46,60,61,62]. Residue 82 is located within an alpha-helix that sits at the base of the NPC1 binding pocket. Residue 544 is located within the internal fusion loop of GP_2_. The effects of these two changes have been described as reducing the threshold for NPC1 activation of the conformational change in GP_2_ that mediates membrane fusion and EBOV infection [46]. It has also been reported that the changes resulting from T544I are not related with cathepsin B proteolytic activity [61].

In our hands, testing with replication competent EBOV with genotypes representing all four potential combinations of GP82A or GP82V with GP544T or GP544I, we found that the effect of the cell culture adaptation T544I is significantly more profound than the A82V change. The T544I change results in a significant decrease in the sensitivity of the mutants carrying this change to 3.47 and related compounds. On the other hand, the A82V change has minor effects (in relative terms) but also appears to decrease sensitivity to 3.47 compounds. Importantly, the magnitude of the change is very different for both changes, which may be explained by the fact that the T544I change is induced artificially during cell culture of several different isolates of EBOV in Vero E6 cells. This underlies the need to carefully select virus challenge stocks to minimize the potential effect of cell culture adaptation in the development of MCMs. 

In addition to EBOV/Mak mutants, we also generated full genome clones and successfully rescued specific isolates of the EBOV variants EBOV/Itu and EBOV/Tum, responsible for recent outbreaks in COD. EBOV/Itu and EBOV/Tum are 98% identical at the nucleotide level. The EBOV/Itu and EBOV/Tum reverse-genetics systems will allow us to investigate whether specific differences in the viral genomes can help explain the vastly different outbreaks caused by these two viruses. As observed in the 2018–2020 EVD outbreak in Nord-Kivu Province, COD, early genomic characterization revealed several synapomorphic (clade-defining) amino acid changes. Notably, a valine to alanine change at position 75 (V75A) in the EBOV GP_1,2_ receptor binding domain showed evidence for positive selection and was estimated to have emerged around October 2018. From 3 January 2019 to the end of the outbreak in April 2020, the V75A change was found in approximately 80% of the 522 genomes sequenced. Considering the V75A changes’s proximity to the A82V change characterized in the EBOV/Mak variant and dominance in consensus genomes, the V75A change may also play a role in infectivity and should be further investigated (Figure 2).

## 5. Conclusions

High-throughput sequencing technologies have facilitated the rapid recovery of filovirus genome sequences during outbreaks. In this work, the use of genome sequence data, coupled with de novo DNA synthesis, has demonstrated the utility of a reverse-genomics approach for generating completely synthetic virus stocks. These synthetic virus stocks can help advance MCM development and be used to investigate virus variants of interest that would otherwise be difficult to obtain or unavailable for study.

## Disclaimer

The views and conclusions contained in this document are those of the authors and should not be interpreted as necessarily representing the official policies, either expressed or implied, of the U.S. Department of Defense, U.S. Department of Health and Human Services, U.S. Department of the Army, or of the institutions and companies affiliated with the authors. In no event shall any of these entities have any responsibility or liability for any use, misuse, inability to use, or reliance upon the information contained herein. The U.S. departments do not endorse any products or commercial services mentioned in this publication.

## Figures and Tables

**Table 1:** Recombinant EBOV/Mak genotypes used in this study as templates for the on-demand pipeline.

Designation for This Study	Genotype Compared to EBOV/ Mak-G3864.1 Sequence 412	Characteristics	GenBank Accession Number
rEBOV/Mak-G3864.1 mutant r412 (parental)	C3579A (*VP35* gene, silent)		MN975530
rEBOV/Mak-G3864.1 mutant r414	C1498T (*NP* gene, silent)	Detected during a flare-up of seven EVD cases that occurred in June and July of 2015 in Liberia [41]. The genome sequence of this virus indicated a markedly reduced rate of genome evolution compared to previously circulating viruses.	MN975531
	G9355A (nonsense, truncation of last 7 amino acids of VP30)		
	A10130G (*VP24* gene UTR)		
rEBOV/Mak-G3864.1 mutant r424	T3008C (*NP* gene UTR)	The double *NP* mutation was first identified in viruses from September 2014 and affects expression of NP in an EBOV minigenome assay [42].	MN975532
	T3011C (*NP* gene UTR)		
	C3579A (*VP35* gene, silent)		
rEBOV/Mak-G3864.1 mutant r440	C6283T (GP_1,2_, V82A)	EBOV/Mak genome sequences obtained at the very beginning of the 2013–2016 EVD were all of the 82A genotype, but the 82V genotype quickly emerged and >90% of all of the sequenced EBOV genomes from the outbreak are of the 82V genotype [39]. The A82V change enhances EBOV infection in nonhuman primate cells [26,27,28,43,44,45,46], but it does not appear to alter virulence in an interferon alpha receptor knock-out (IFNAR^−/−^) laboratory mouse model and a rhesus monkey (*Macaca mulatta*) model of EVD [47].	MN975533
	C3579A (*VP35* gene, silent)		
rEBOV/Itu mutant r865	N/A		MN975534
rEBOV/Tum mutant r866	N/A		MN975535

EBOV = Ebola virus; rEBOV = recombinant EBOV; EBOV/Mak = EBOV variant Makona; EBOV/Tum = EBOV variant Tumba; EBOV/Itu = EBOV variant Ituri; *VP* = viral protein gene; VP = viral protein; *NP* = nucleoprotein; GP_1,2_ = glycoprotein; UTR = untranslated region; EVD = Ebola virus disease.

**Table 2:** Minor variants detected in rEBOV/Mak-G3864.1 mutant “r412” stocks.

Sample	Variants >5%	% 8U
HeLa ^1^	G7692A (31.5%): GP_1,2_ D552N	0.8
HeLa ^2^	T331C (26%) in *NP* UTR	1.8
	ins11514A (38.9%) in *VP24*/*L* UTR	
Huh7D-12/T7	none	1.1
Vero E6 (1)	none	0.9
Vero E6 (2)	A670G (25.8%): NP Q67R	9.1
	C16881T (7.9%): L T1767I	
Huh7 plus T7 *	none	1.2
Huh7D-12/T7 plus T7 *	none	1.4
VeroE6 plus T7 *	A4943T (100%): VP40 Q155L	2.4
	A9124C (68.1%): VP30 E205D	

^1^ First experiment; ^2^ Second, repeated experiment; * These cell lines do not express T7; therefore, a T7 RNA polymerase expressing plasmid was included in the transfection. rEBOV = recombinant EBOV; EBOV = Ebola virus; EBOV/Mak = EBOV variant Makona; GP_1,2_ = glycoprotein; *NP* = nucleoprotein gene; NP = nucleoprotein; *VP* = viral protein gene; VP = viral protein; *L* = large protein gene; L = large protein.

**Table 3:** Antiviral activity of EBOV entry inhibitor 3.47 and its analogs.

Virus	Phenotype	Compound ID	EC_50_ (μM)	SD	EC_90_	CC_50_ (μM)	SI
EBOV/Mak-r440	82A 544T	3.47	0.007	0.0004	0.02	>10	>1423
EBOV/Mak-r412	82V 544T		0.01	0.001	0.08	>10	>1014
EBOV/Mak-201403261	82V 544I		0.04	0.008	1.67	>10	>248
EBOV/May-tc	82A 544I		0.01	0.005	0.74	>10	>792
EBOV/Mak-r440	82A 544T	1412	0.15	0.12	5.65	>20	>131
EBOV/Mak-r412	82V 544T		0.77	0.23	16	>20	>26
EBOV/Mak-201403261	82V 544I		2.93	1.21	79.7	>20	>7
EBOV/May-tc	82A 544I		2.64	1.14	106	>20	>8
EBOV/Mak-r440	82A 544T	1413	0.08	0.02	0.68	>20	>266
EBOV/Mak-r412	82V 544T		0.24	0.06	4.03	>20	>85
EBOV/Mak-201403261	82V 544I		0.85	0.33	27,8	>20	>24
EBOV/May-tc	82A 544I		0.76	0.27	18.7	>20	>26
EBOV/Mak-r440	82A 544T	1466	0.04	0.007	0.14	>20	>497
EBOV/Mak-r412	82V 544T		0.13	0.02	1.39	>20	>159
EBOV/Mak-201403261	82V 544I		0.39	0.17	24.4	>20	>52
EBOV/May-tc	82A 544I		0.30	0.13	11.1	>20	>67
EBOV/Mak-r440	82A 544T	1476	0.001	0.0004	0.05	>10	>14,169
EBOV/Mak-r412	82V 544T		0.002	0.001	0.17	>10	>5985
EBOV/Mak-201403261	82V 544I		0.005	0.002	0.4	>10	>2018
EBOV/May-tc	82A 544I		0.002	0.002	0.37	>10	>4262

EBOV = Ebola virus; EBOV/Mak = EBOV variant Makona; EBOV/May-tc = tissue-culture adapted EBOV/Yam-May; ID = identification; EC_50_ = half-maximal effective concentration; SD = standard deviation; EC_90_ = maximal effective concentration; CC_50_ = cytotoxic concentration; SI = selective index.

## Data Availability

Plasmid and viral sequences are all publically available on NCBI’s GenBank repository.
